# Supplementary material for: Disentangling five dimensions of animacy in human brain and behaviour
Source: Commun Biol. 2022 Nov 14;5:1247. doi: 10.1038/s42003-022-04194-y (PMC9663603; doi:10.1038/s42003-022-04194-y)
Supplement: Supplementary file 2 — Supplementary Information [file 42003_2022_4194_MOESM2_ESM.docx]

**Supplementary Information**

**Disentangling five dimensions of animacy in human brain and behaviour**

Kamila M. Jozwik, Elias Najarro, Jasper J.F. van den Bosch, Ian Charest, Radoslaw M. Cichy* and Nikolaus Kriegeskorte*

| animal robot |
| --- |
| animal robot Boston dynamics |
| animal sculpture |
| animal shadow |
| ball |
| bear rug |
| bike |
| bird clock |
| boomerang |
| bus |
| car |
| carnivorous plant |
| cat |
| cells |
| chimpanzee |
| clouds |
| clownfish |
| colony insects |
| comet |
| coral |
| corpse |
| cow |
| disabled person with motor neuron disease |
| dog |
| doll |
| domino falling |
| eyeball |
| fire |
| flowers |
| frisbee |
| frog |
| fungi |
| gambling machine |
| geyser |
| giraffe |
| hammer |
| helicopter |
| household robot |
| human body |
| human embryo |
| human face |
| human fetus |
| human heart |
| human in coma |
| human robot |
| human robot Boston dynamics |
| human sculpture |
| human shadow |
| human with prosthetics |
| humanoid robot |
| imprisoned animal |
| imprisoned human |
| industrial robot |
| jack in box |
| Jenga tower |
| key |
| kite |
| ladybug |
| lava |
| lobster |
| meat |
| mechanical bull |
| microwave |
| oyster |
| pelican |
| photocopier |
| pig |
| plane |
| plant |
| plush robot |
| plush toy |
| puppet |
| roasted animal |
| rock |
| rolling dice |
| salamander |
| sea sponge |
| sea urchin |
| skeleton |
| small baby |
| small baby face |
| smoke |
| snake |
| soil |
| starfish |
| still water |
| stingray |
| storm |
| toy horse with wheels |
| train |
| tree |
| trophy |
| turtle |
| volcano eruption |
| warbler |
| washing machine |
| waves |
| weeds |
| wheel |
| whirlwind |

**Supplementary Table 1. Object categories from step 1 of stimulus selection procedure - labels generated by participants when filling animacy dimension grid combinations.**


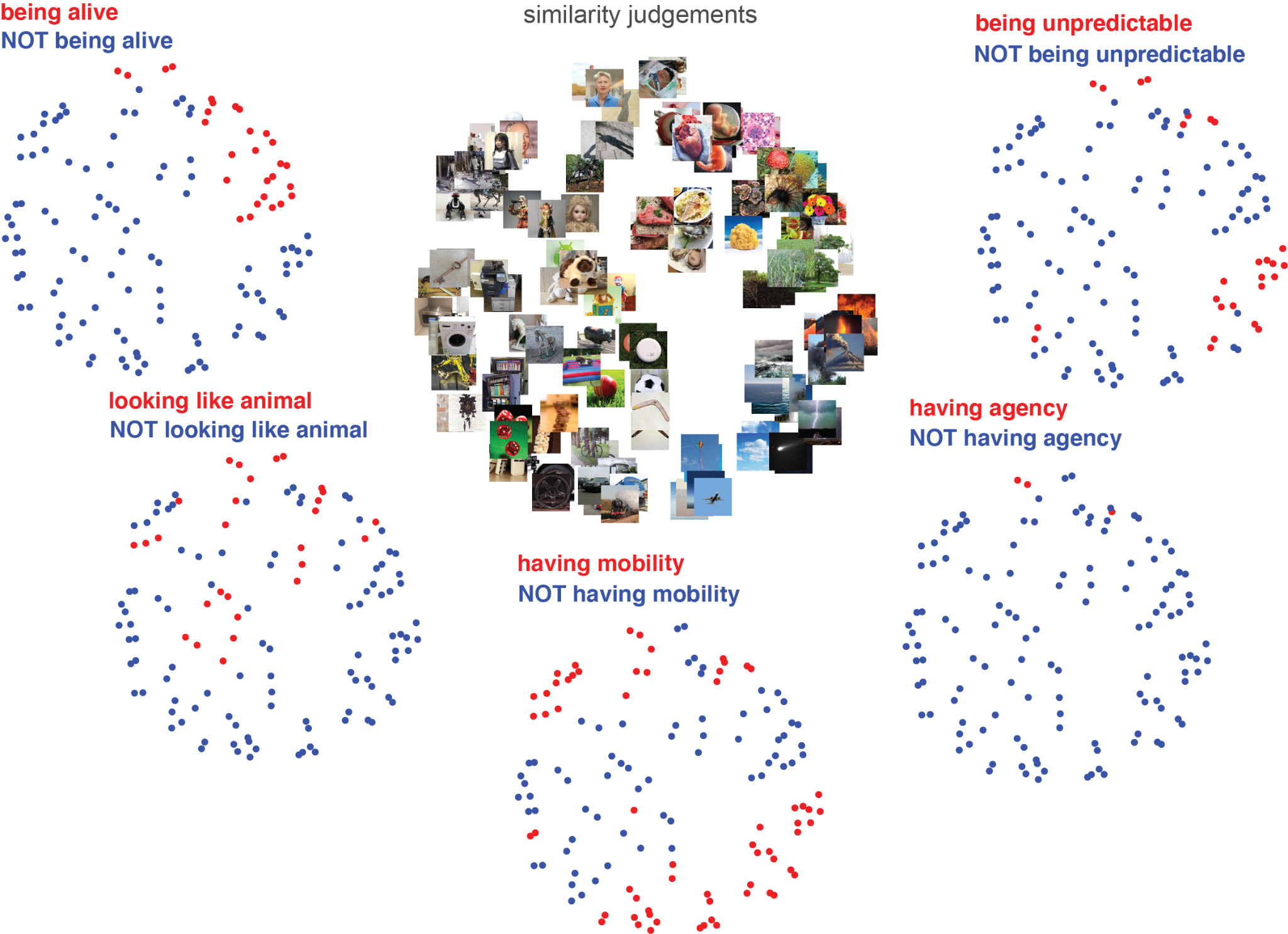


**Supplementary Figure 1.** Multidimensional scaling plot of similarity judgements (mean across participants, with metric stress criterion) colour coded based on the selected dimensions of animacy ratings.

**
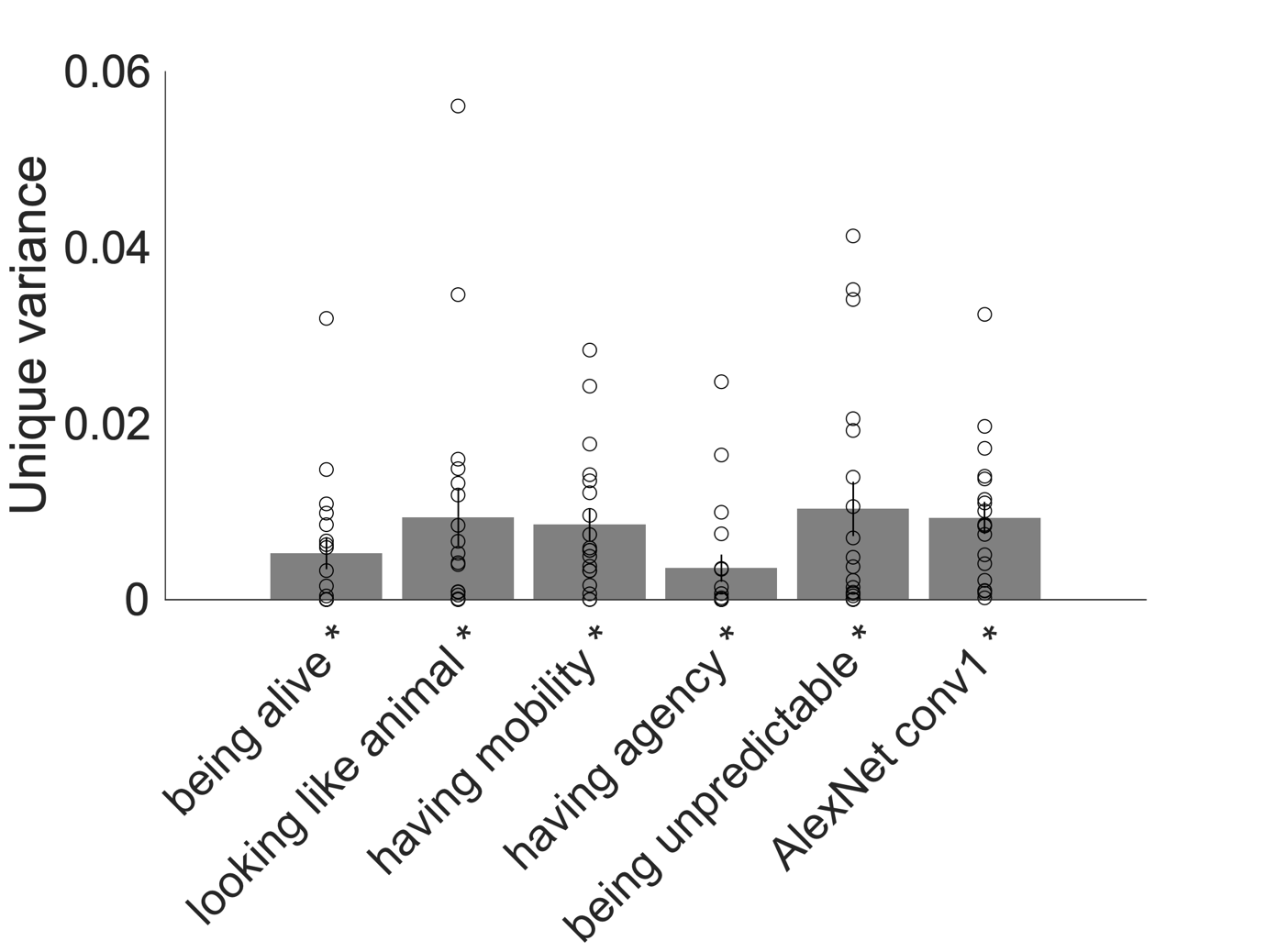
**

**Supplementary Figure 2. Unique variance analysis for similarity judgements including the first convolutional layer of AlexNet (AlexNet conv1).** Bars show the unique variance in similarity judgements RDMs. For each animacy dimension m, the unique variance was computed by subtracting the total variance explained by the reduced GLM (excluding the dimension of interest) from the total variance explained by the full GLM. Specifically, for dimension m, we fit GLM on X = "all dimensions but m" and Y = data, then we subtract the resulting R2 from the total R2 (fit GLM on X = "all dimensions" and Y = data). We used non-negative least squares to find optimal weights. A significant unique variance is indicated by an asterisk (one-sided Wilcoxon signed-rank test, p < 0.05 corrected). The error bars show the standard error of the mean based on single-participant unique variance. Circles show single-participant unique variance.

**
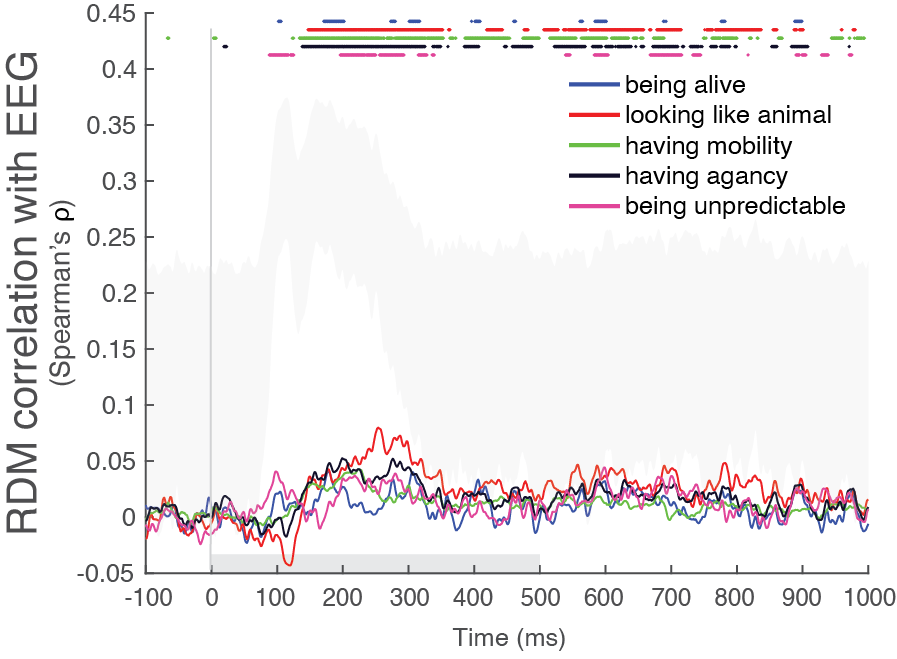
**

**Supplementary Figure 3.** Animacy dimension RDM comparison with EEG RDMs across time with displayed noise ceiling. Lines show the correlation between the EEG RDMs and each animacy dimension RDM. A significant correlation is indicated by a horizontal line above the graph (one-sided Wilcoxon signed-rank test, p < 0.05 corrected). The grey horizontal bar on the x axis indicates the stimulus duration. The grey area represents the noise ceiling, which indicates the expected performance of the true model given the noise in the data. The selected dimensions of animacy do not reach the noise ceiling, indicating that it leaves unexplained some of the variance that is reliable across individual observers.

**
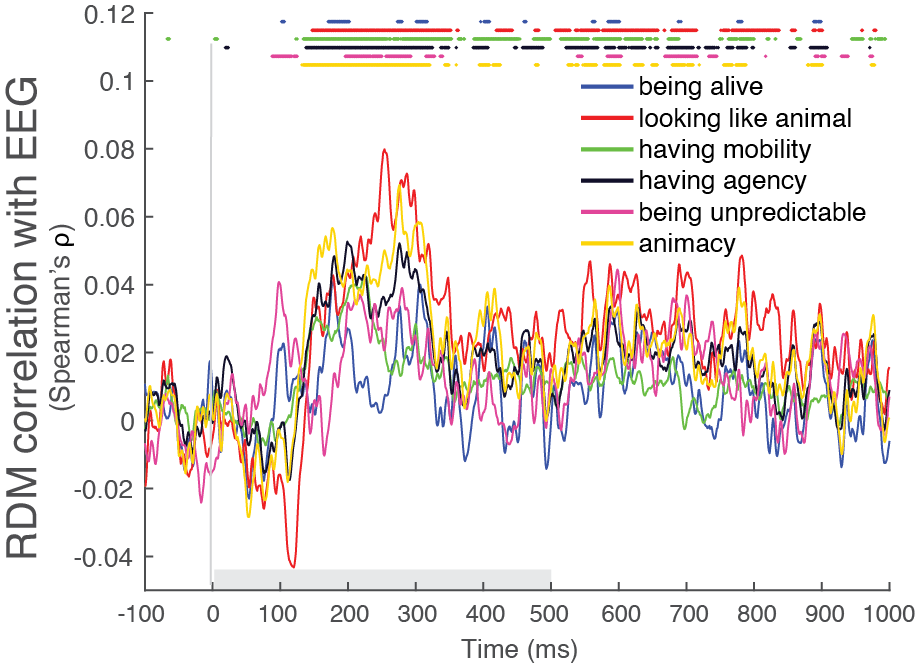
**

**Supplementary Figure 4.** Animacy dimension RDM comparison with EEG RDMs across time including general animacy ratings. Lines show the correlation between the EEG RDMs and each animacy dimension RDM. A significant correlation is indicated by a horizontal line above the graph (one-sided Wilcoxon signed-rank test, p < 0.05 corrected). The grey horizontal bar on the x axis indicates the stimulus duration.

**
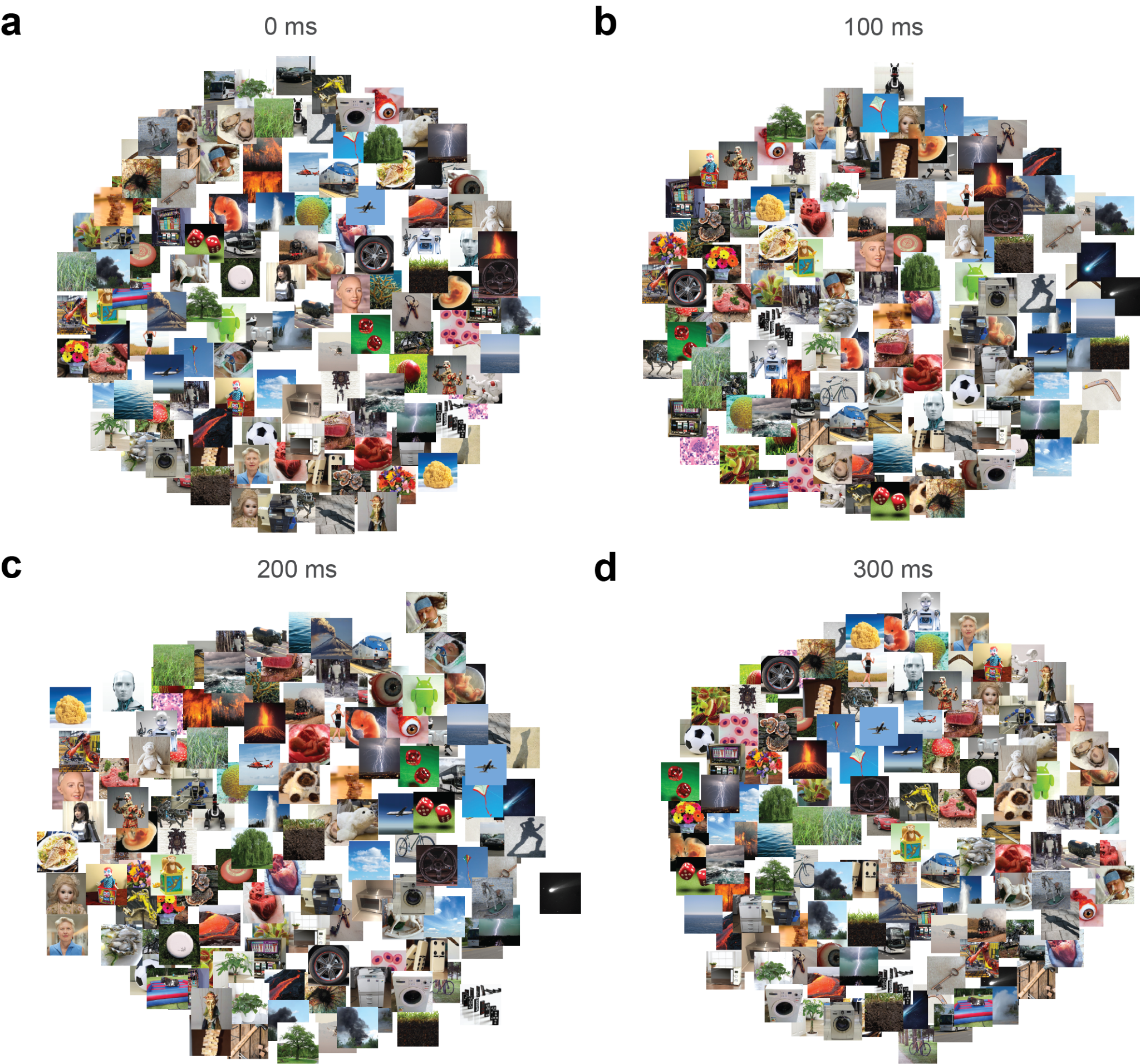
**

**Supplementary Figure 5.** Multidimensional scaling plots at different EEG timepoint RDMs (mean across participants, with metric stress criterion).


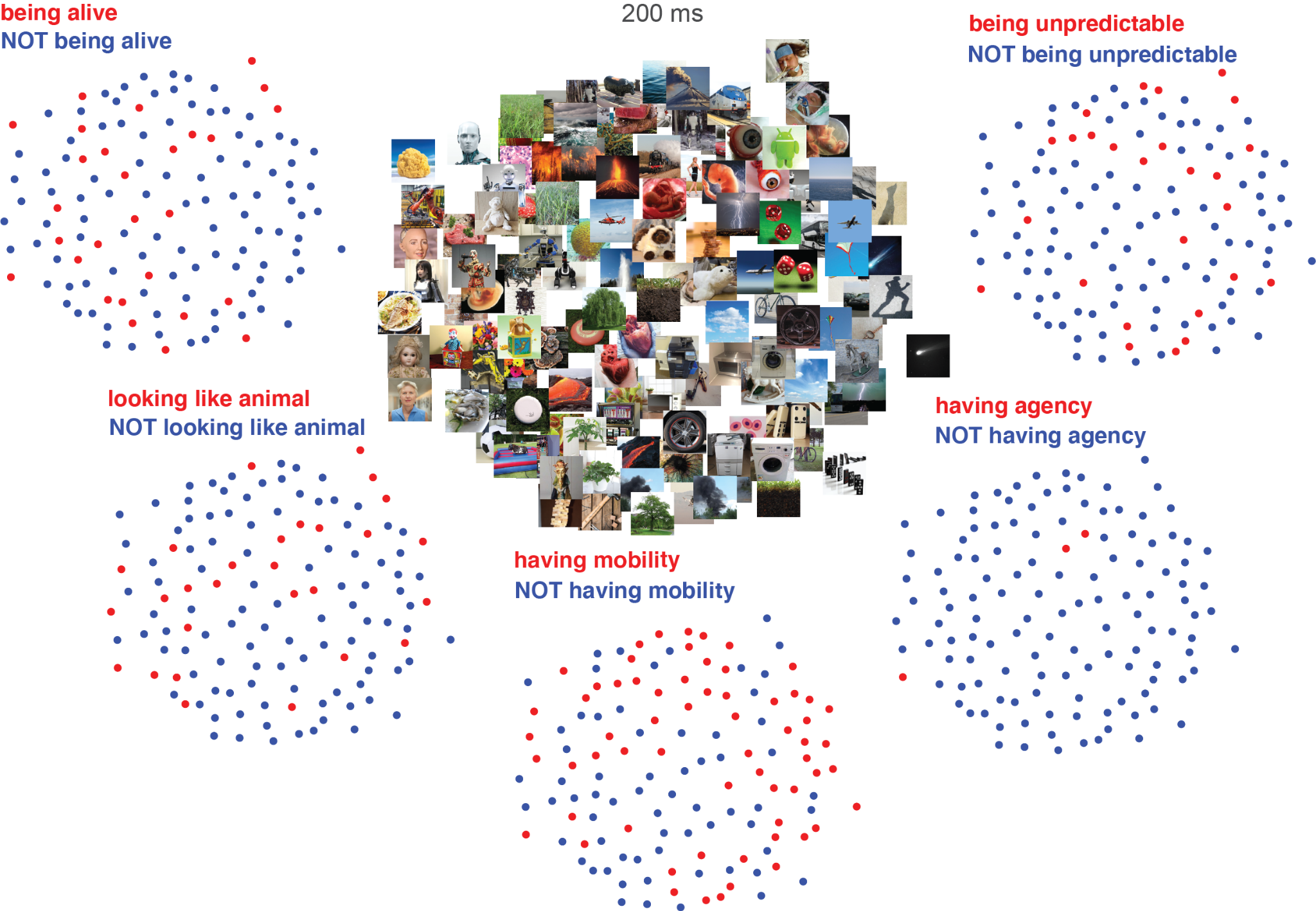


**Supplementary Figure 6.** Multidimensional scaling plots of EEG RDM at 200 ms (mean across participants, with metric stress criterion) colour coded based on the selected dimensions of animacy ratings.

**
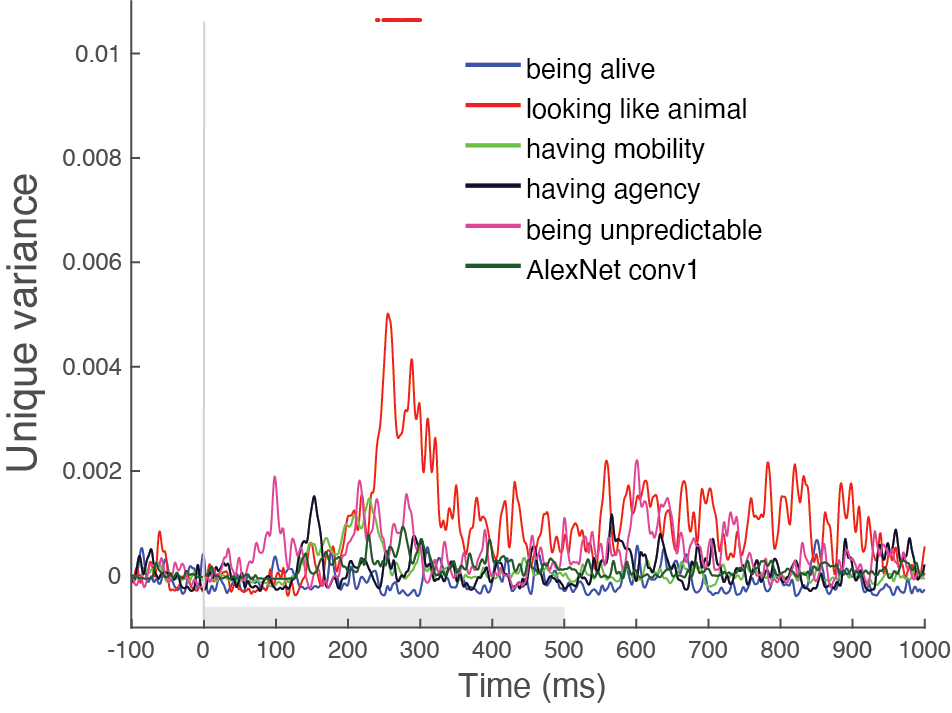
**

**Supplementary Figure 7.** Unique variance of each animacy dimension and the first convolutional layer of AlexNet (AlexNet conv1) in explaining EEG RDMs. For each animacy dimension, the unique variance is computed by subtracting the total variance explained by the reduced GLM (excluding the animacy dimension of interest) from the total variance explained by the full GLM, using non-negative least squares to find optimal weights. A significant unique variance is indicated by a horizontal line above the graph (one-sided Wilcoxon signed-rank test, p < 0.05 corrected). The grey horizontal bar on the x axis indicates the stimulus duration.

**
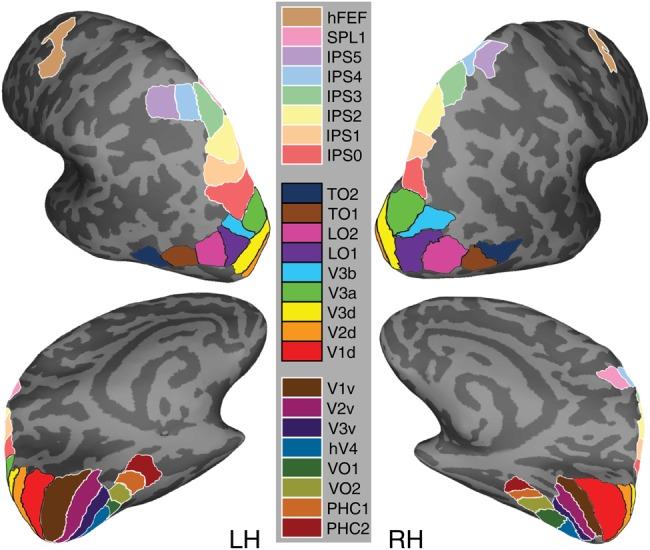
**

**Supplementary Figure 8.** ROI location in ventral and dorsal streams. Reproduced with permission from Wang et al., 2015 (left hemisphere (LH) and right hemisphere (RH)). This study investigated visual representations in ventral (v) and dorsal (d) streams.

**­
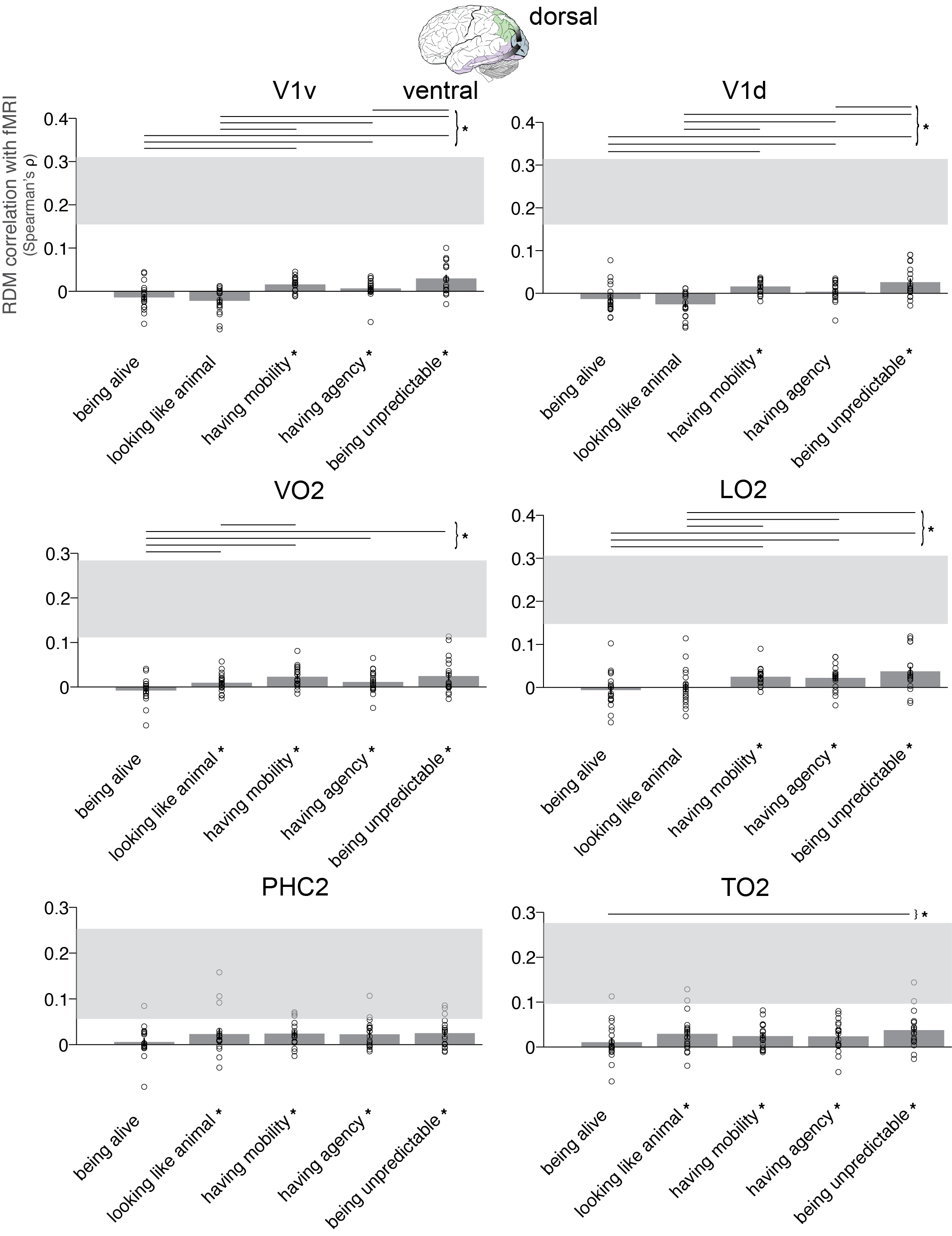
**

**Supplementary Figure 9.** Animacy dimension RDM comparisons with fMRI ROI RDMs with displayed noise ceiling. Bars show the correlation between each animacy dimension RDM with fMRI ROI RDMs. We selected ROIs across the ventral (V1v, VO2, PHC2) and dorsal (V1d, LO2, TO2) visual streams. A significant correlation is indicated by an asterisk (one-sided Wilcoxon signed-rank test, p < 0.05 corrected). Error bars show the standard error of the mean based on single-participant correlations, i.e., correlations between the single-participant ROI RDMs and animacy dimension RDM. Circles show single-participant correlations. The grey bar represents the noise ceiling, which indicates the expected performance of the true model given the noise in the data. Horizontal lines show significant pairwise differences between model (here dimensions of animacy) performance (p < 0.05, FDR corrected across all comparisons), an asterisk to the right of horizontal lines indicates their significance.

**
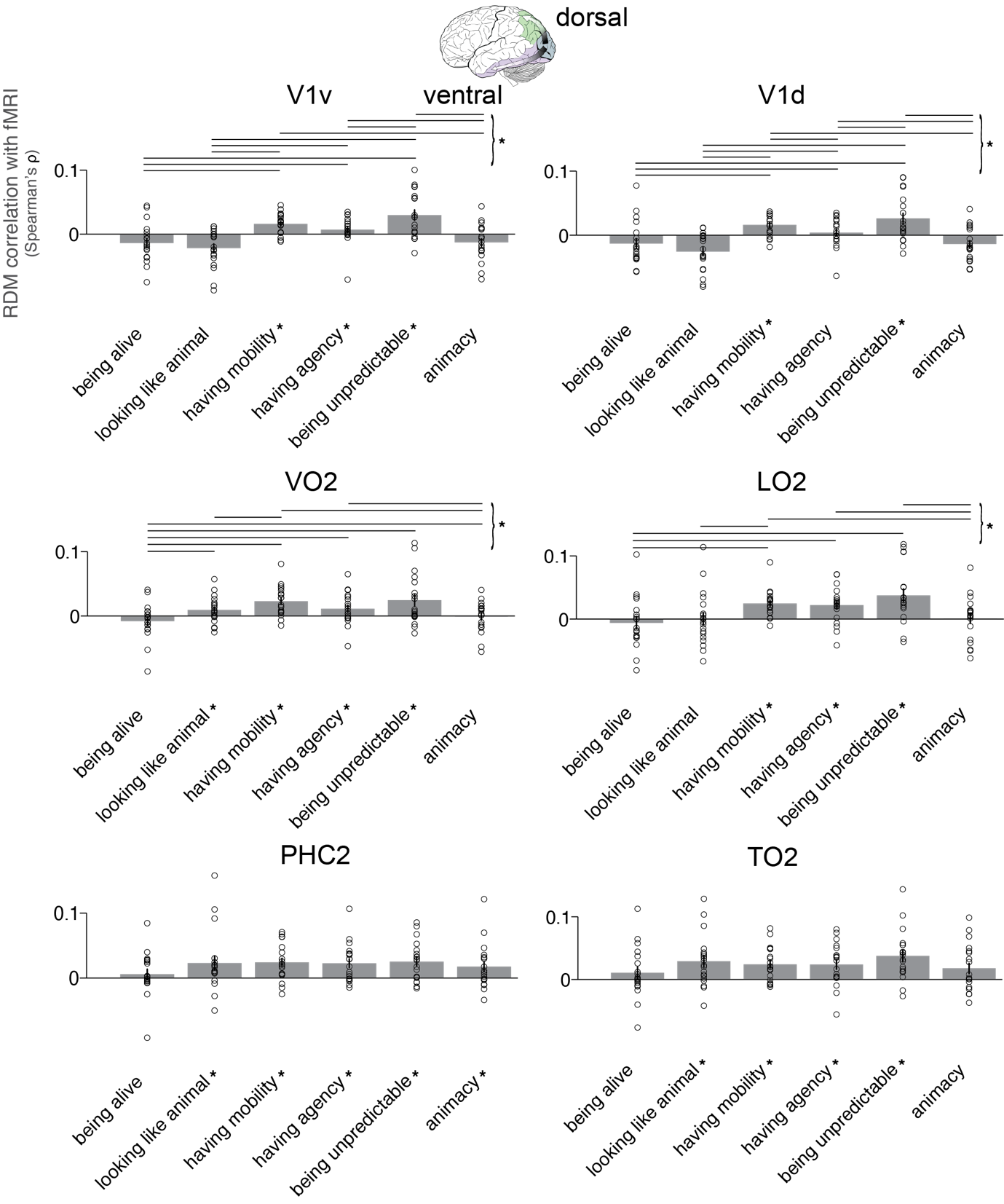
­­­**

**Supplementary Figure 10.** Animacy dimension RDM comparisons with fMRI ROI RDMs including general animacy ratings. Bars show the correlation between each animacy dimension RDM with fMRI ROI RDMs. A significant correlation is indicated by an asterisk (one-sided Wilcoxon signed-rank test, p < 0.05 corrected). Error bars show the standard error of the mean based on single-participant correlations, i.e., correlations between the single-participant ROI RDMs and animacy dimension RDM. Circles show single-participant correlations. Horizontal lines show significant pairwise differences between model performance (p < 0.05, FDR corrected across all comparisons), an asterisk to the right of horizontal lines indicates their significance.


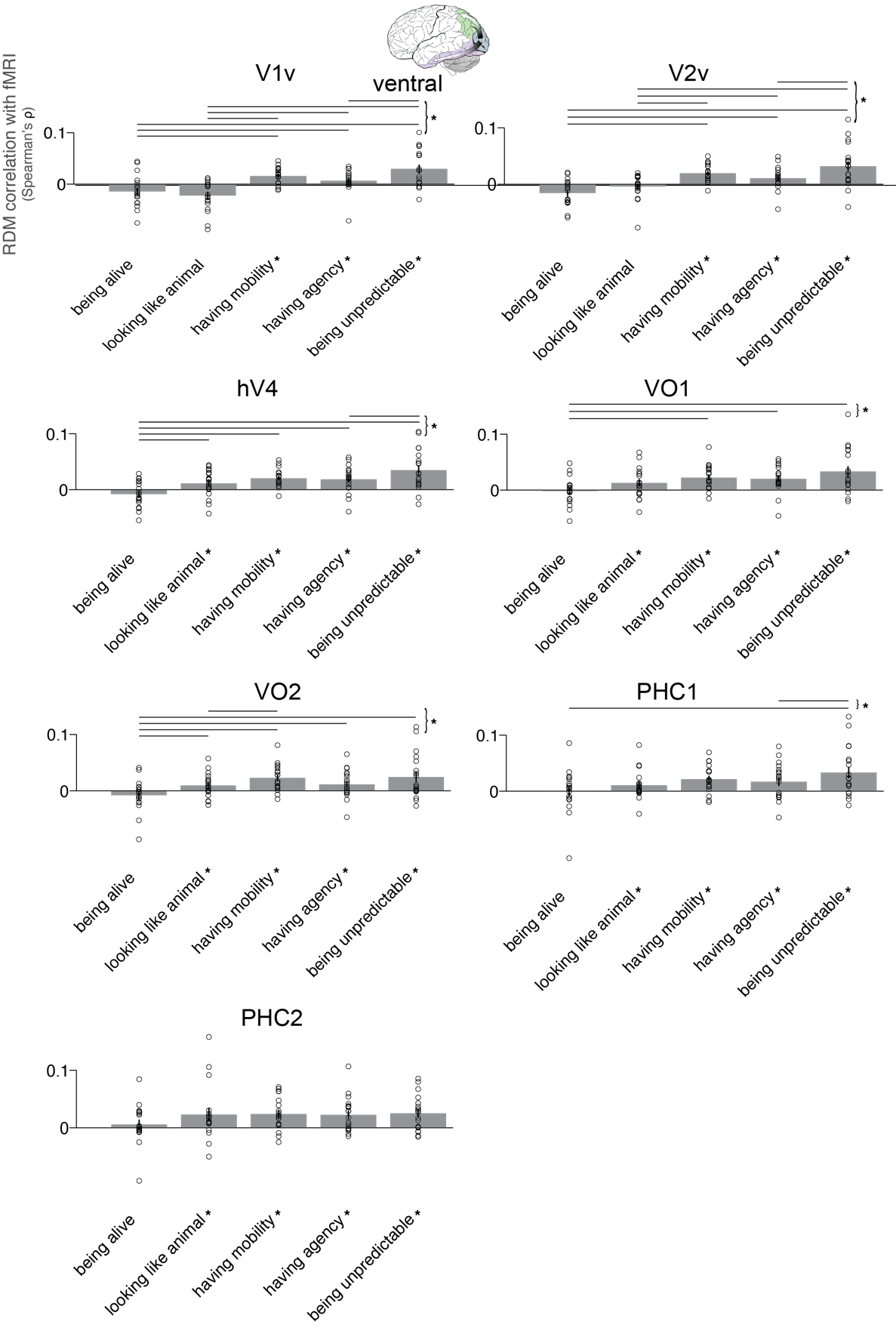


**Supplementary Figure 11.** Animacy dimension RDM comparisons with fMRI ROI RDMs across all ventral stream ROIs based on Wang et al., 2015 (see Supplementary Figure 8 for ROI locations). The ventral stream ROIs included V1v, V2v, hV4, VO1, VO2, PHC1 and PHC2 (V3v was omitted as there were not enough voxels in this ROI). Bars show the correlation between each animacy dimension RDM with fMRI ROI RDMs. A significant correlation is indicated by an asterisk (one-sided Wilcoxon signed-rank test, p < 0.05 corrected). Error bars show the standard error of the mean based on single-participant correlations, i.e., correlations between the single-participant ROI RDMs and animacy dimension RDM. Circles show single-participant correlations. Horizontal lines show significant pairwise differences between model performance (p < 0.05, FDR corrected across all comparisons), an asterisk to the right of horizontal lines indicates their significance.

**_­­­
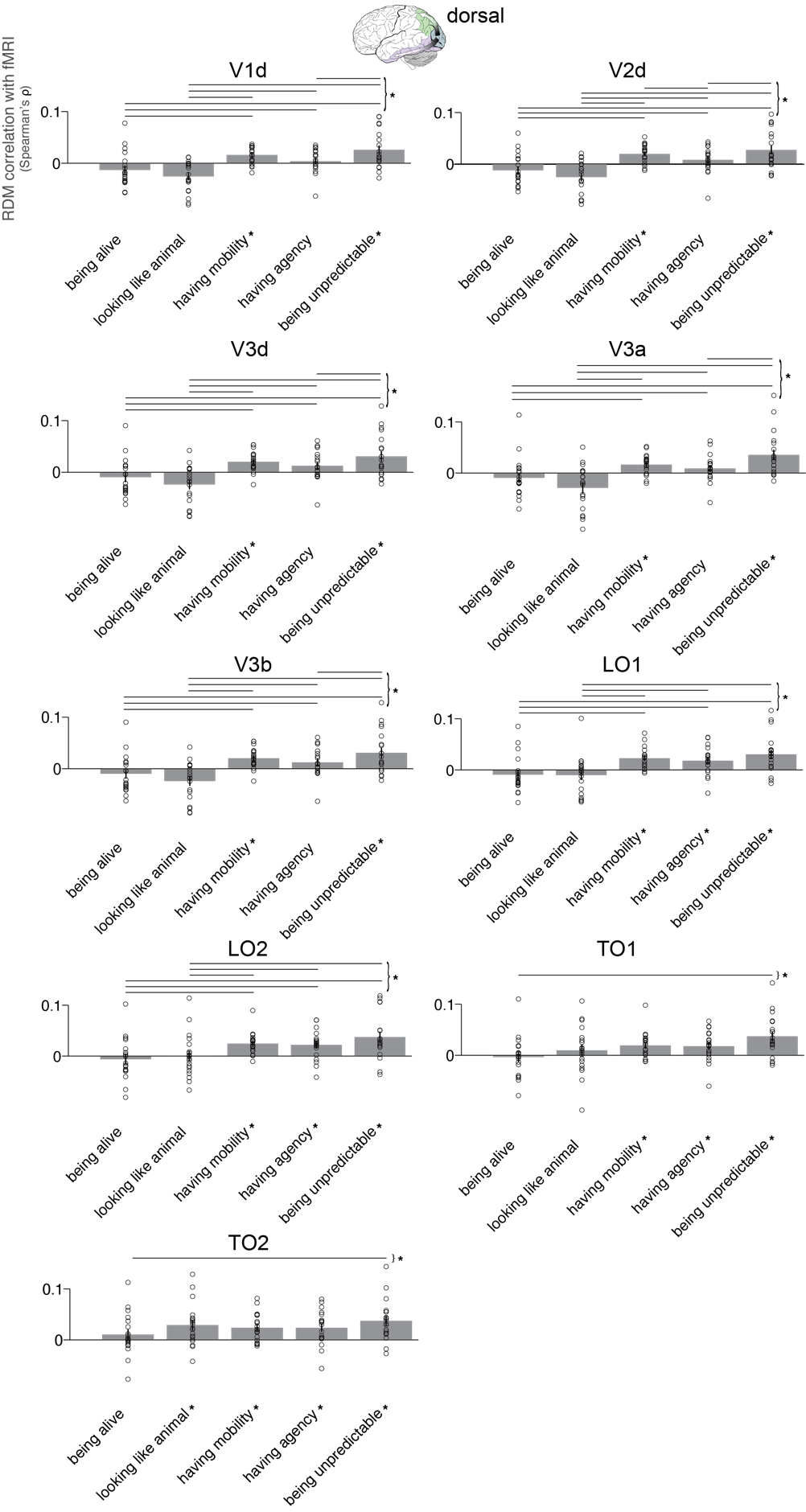
_**

**_­­­_Supplementary Figure 12.** Animacy dimension RDM comparisons with fMRI ROI RDMs across all dorsal stream ROIs based on Wang et al., 2015 (see Supplementary Figure 8 for ROI locations). The dorsal stream ROIs included V1d, V2d, V3d, V3a, V3b, LO1, LO2, TO1 and TO2. Bars show the correlation between each animacy dimension RDM with fMRI ROI RDMs. A significant correlation is indicated by an asterisk (one-sided Wilcoxon signed-rank test, p < 0.05 corrected). Error bars show the standard error of the mean based on single-participant correlations, i.e., correlations between the single-participant ROI RDMs and animacy dimension RDM. Circles show single-participant correlations. Horizontal lines show significant pairwise differences between model performance (p < 0.05, FDR corrected across all comparisons), an asterisk to the right of horizontal lines indicates their significance.

**
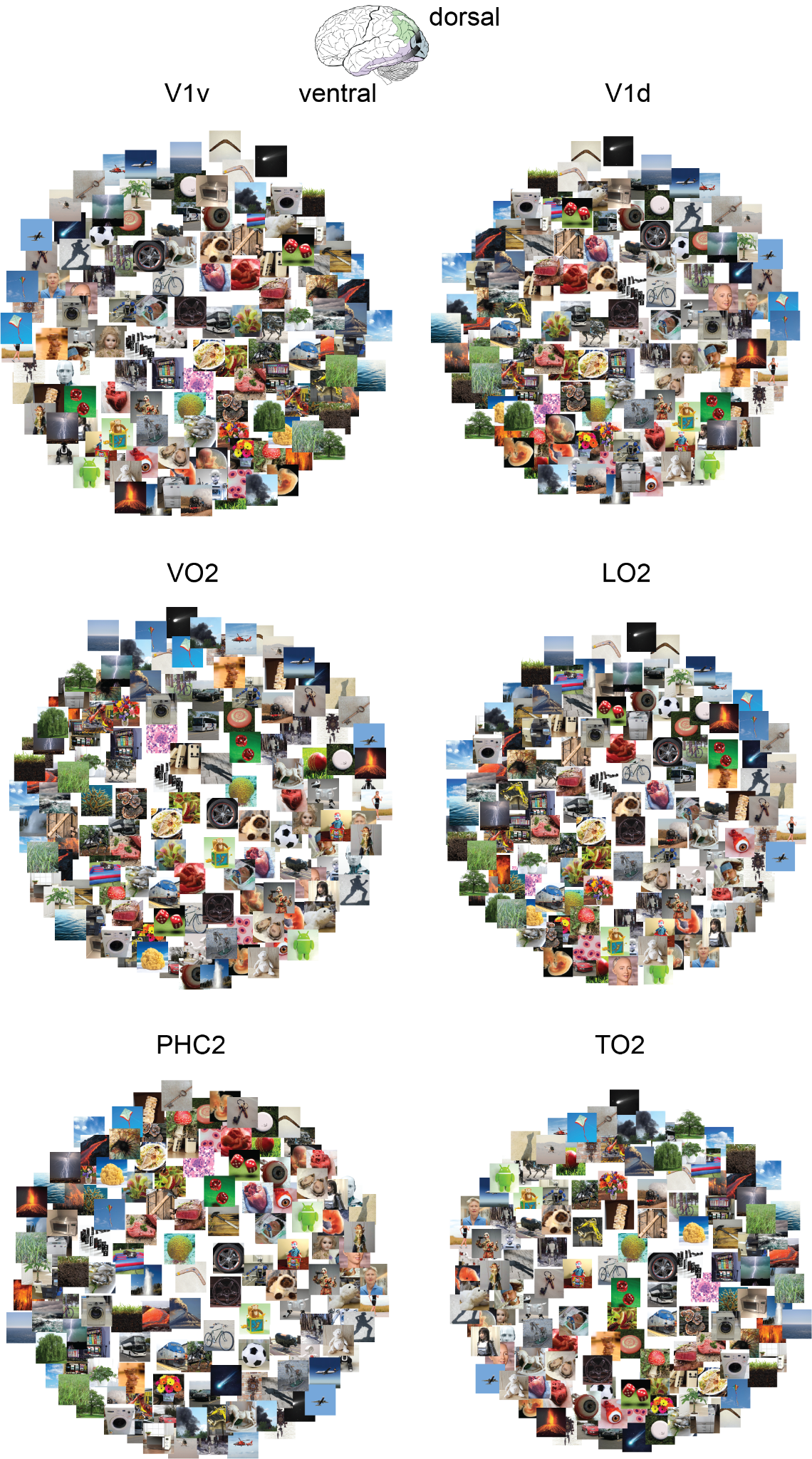
**

**Supplementary Figure 13.** Multidimensional scaling plots of fMRI ROI RDMs (mean across participants, with metric stress criterion).

**
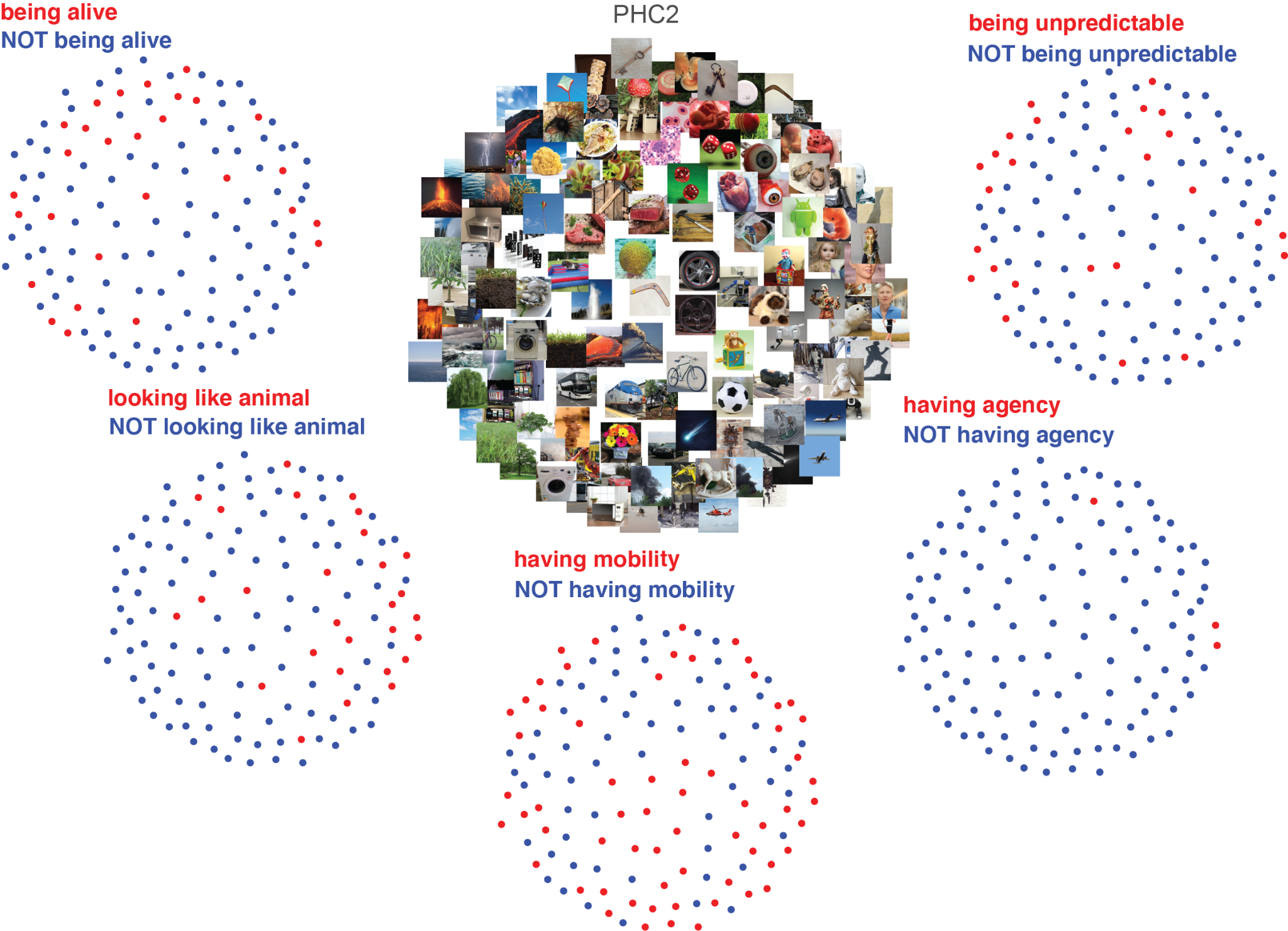
**

**Supplementary Figure 14.** Multidimensional scaling plots of PHC2 fMRI ROI RDM (mean across participants, with metric stress criterion) colour coded based on the selected dimensions of animacy ratings.


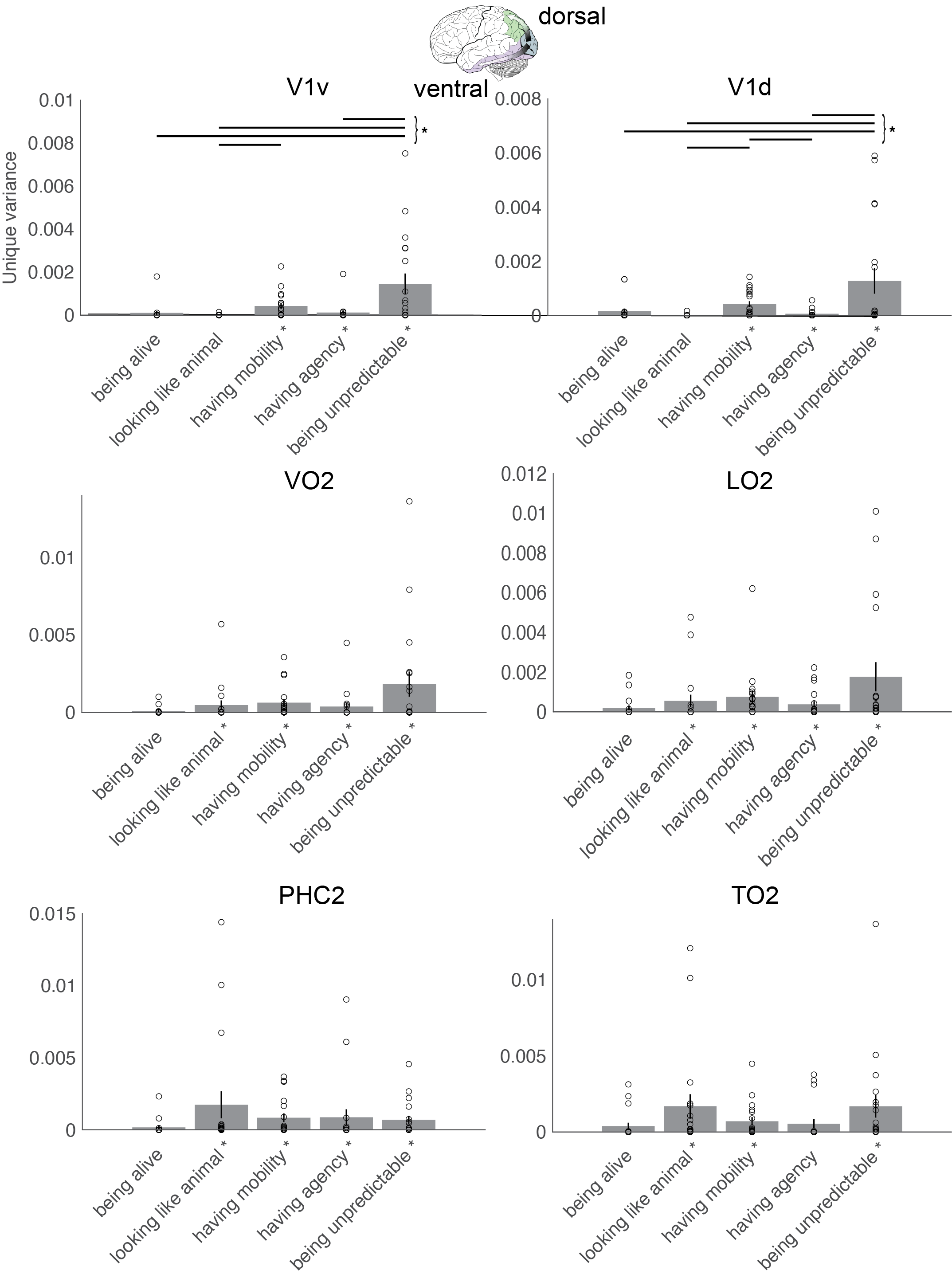


**Supplementary Figure 15. Unique variance analysis for fMRI responses.** Bars show the unique variance in fMRI ROI RDMs. We selected ROIs across the ventral (V1v, VO2, PHC2) and dorsal (V1d, LO2, TO2) visual streams. For each animacy dimension m, the unique variance was computed by subtracting the total variance explained by the reduced GLM (excluding the dimension of interest) from the total variance explained by the full GLM. Specifically, for dimension m, we fit GLM on X = "all dimensions but m" and Y = data, then we subtract the resulting R2 from the total R2 (fit GLM on X = "all dimensions" and Y = data). We used non-negative least squares to find optimal weights. A significant unique variance is indicated by an asterisk (one-sided Wilcoxon signed-rank test, p < 0.05 corrected). The error bars show the standard error of the mean based on single-participant unique variance. Circles show single-participant unique variance. Horizontal lines show significant pairwise differences between model performance (p < 0.05, FDR corrected across all comparisons), an asterisk to the right of horizontal lines indicates their significance.


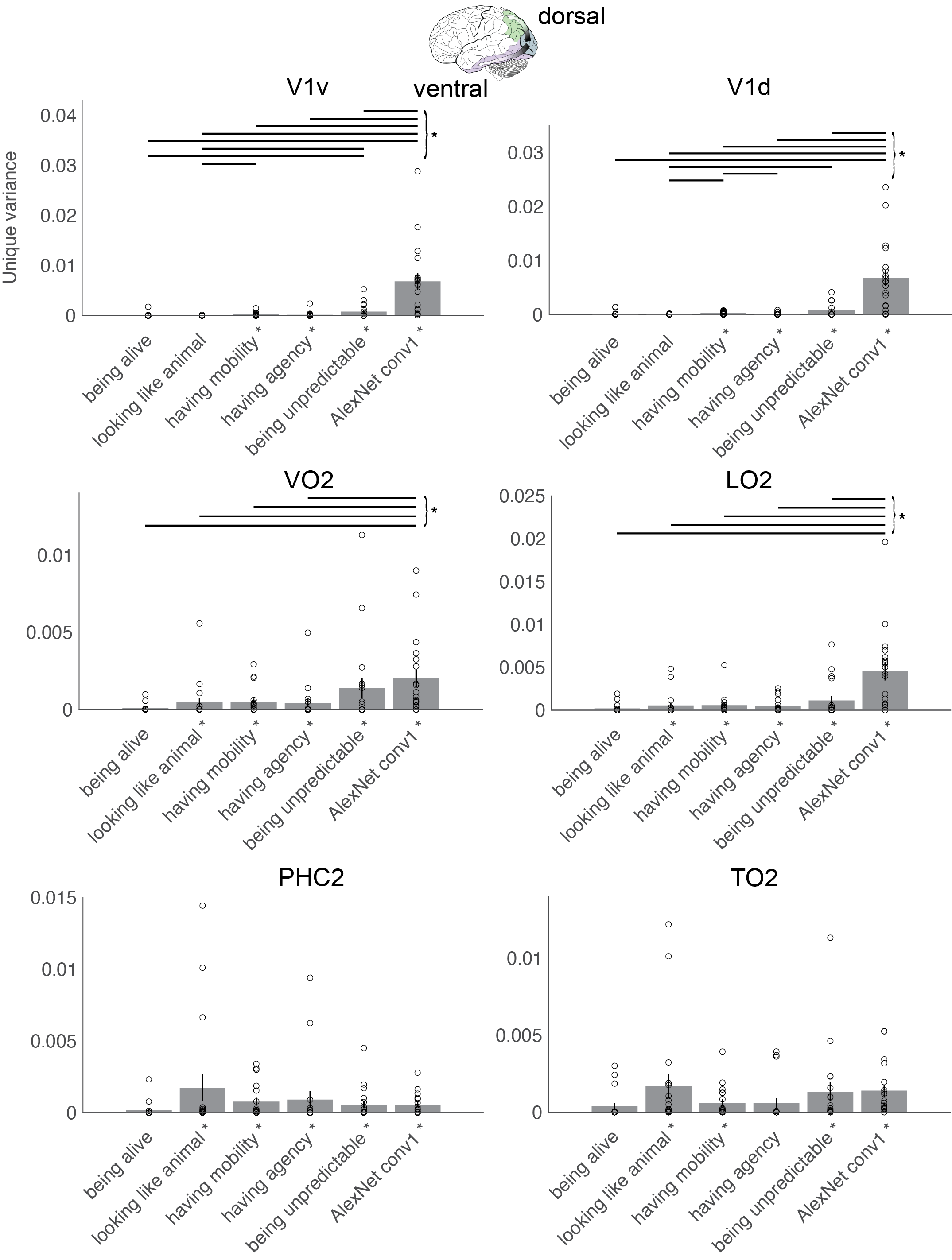


**Supplementary Figure 16. Unique variance analysis for fMRI responses including the first convolutional layer of AlexNet** **(AlexNet conv1).** Bars show the unique variance in fMRI ROI RDMs. We selected ROIs across the ventral (V1v, VO2, PHC2) and dorsal (V1d, LO2, TO2) visual streams. For each animacy dimension m, the unique variance was computed by subtracting the total variance explained by the reduced GLM (excluding the dimension of interest) from the total variance explained by the full GLM. Specifically, for dimension m, we fit GLM on X = "all dimensions but m" and Y = data, then we subtract the resulting R2 from the total R2 (fit GLM on X = "all dimensions" and Y = data). We used non-negative least squares to find optimal weights. A significant unique variance is indicated by an asterisk (one-sided Wilcoxon signed-rank test, p < 0.05 corrected). The error bars show the standard error of the mean based on single-participant unique variance. Circles show single-participant unique variance. Horizontal lines show significant pairwise differences between model performance (p < 0.05, FDR corrected across all comparisons), an asterisk to the right of horizontal lines indicates their significance.
