# Supplementary material for: Disentangling five dimensions of animacy in human brain and behaviour
Source: Commun Biol. 2022 Nov 14;5:1247. doi: 10.1038/s42003-022-04194-y (PMC9663603; doi:10.1038/s42003-022-04194-y)
Supplement: Supplementary file 3 — Description of Additional Supplementary Files [file 42003_2022_4194_MOESM3_ESM.pdf]

Description of Additional Supplementary Files

File name: Supplementary Data 1

Description: Source data files for Figures 4b, 4c, 5c, 5d, and 7a.
