# Supplementary material for: Disentangling five dimensions of animacy in human brain and behaviour
Source: Commun Biol. 2022 Nov 14;5:1247. doi: 10.1038/s42003-022-04194-y (PMC9663603; doi:10.1038/s42003-022-04194-y)
Supplement: Supplementary file 5 — Reporting Summary [file 42003_2022_4194_MOESM5_ESM.pdf]

nature research

Corresponding author(s): Kamila Jozwik

Last updated by author(s): Oct 3, 2022

Reporting Summary

Nature Research wishes to improve the reproducibility of the work that we publish. This form provides structure for consistency and transparency in reporting. For further information on Nature Research policies, see our Editorial Policies and the Editorial Policy Checklist.

Statistics

For all statistical analyses, confirm that the following items are present in the figure legend, table legend, main text, or Methods section.

n/a	Confirmed	
☐	☒	The exact sample size (*n*) for each experimental group/condition, given as a discrete number and unit of measurement
☐	☒	A statement on whether measurements were taken from distinct samples or whether the same sample was measured repeatedly
☐	☒	The statistical test(s) used AND whether they are one- or two-sided<br>*Only common tests should be described solely by name; describe more complex techniques in the Methods section.*
☒	☐	A description of all covariates tested
☐	☒	A description of any assumptions or corrections, such as tests of normality and adjustment for multiple comparisons
☐	☒	A full description of the statistical parameters including central tendency (e.g. means) or other basic estimates (e.g. regression coefficient) AND variation (e.g. standard deviation) or associated estimates of uncertainty (e.g. confidence intervals)
☒	☐	For null hypothesis testing, the test statistic (e.g. *F*, *t*, *r*) with confidence intervals, effect sizes, degrees of freedom and *P* value noted<br>*Give P values as exact values whenever suitable.*
☒	☐	For Bayesian analysis, information on the choice of priors and Markov chain Monte Carlo settings
☒	☐	For hierarchical and complex designs, identification of the appropriate level for tests and full reporting of outcomes
☐	☒	Estimates of effect sizes (e.g. Cohen's *d*, Pearson's *r*), indicating how they were calculated

Our web collection on statistics for biologists contains articles on many of the points above.

Software and code

Policy information about availability of computer code

Data collection	Data was collected using MATLAB and JavaScript.
Data analysis	Data was analyzed using MATLAB, SPM 12, and Brainstorm.

For manuscripts utilizing custom algorithms or software that are central to the research but not yet described in published literature, software must be made available to editors and reviewers. We strongly encourage code deposition in a community repository (e.g. GitHub). See the Nature Research guidelines for submitting code & software for further information.

Data

Policy information about availability of data

All manuscripts must include a data availability statement. This statement should provide the following information, where applicable:

- Accession codes, unique identifiers, or web links for publicly available datasets
- A list of figures that have associated raw data
- A description of any restrictions on data availability

The datasets generated during the current study are available from the corresponding author on request.

Field-specific reporting

Please select the one below that is the best fit for your research. If you are not sure, read the appropriate sections before making your selection.

☒ Life sciences ☐ Behavioural & social sciences ☐ Ecological, evolutionary & environmental sciences

For a reference copy of the document with all sections, see nature.com/documents/nr-reporting-summary-flat.pdf

Life sciences study design

All studies must disclose on these points even when the disclosure is negative.

Sample size	19 subjects
Data exclusions	no data exclusion
Replication	19 subjects and repeated 128 images shown in EEG experiment, fMRI experiment, and behavioural experiments
Randomization	no experimental groups, stimuli shown in random order for all the participants in all the experiments
Blinding	no group allocation

Reporting for specific materials, systems and methods

We require information from authors about some types of materials, experimental systems and methods used in many studies. Here, indicate whether each material, system or method listed is relevant to your study. If you are not sure if a list item applies to your research, read the appropriate section before selecting a response.

Materials & experimental systems

n/a	Involved in the study	
☒	☐	Antibodies
☒	☐	Eukaryotic cell lines
☒	☐	Palaeontology and archaeology
☒	☐	Animals and other organisms
☐	☒	Human research participants
☒	☐	Clinical data
☒	☐	Dual use research of concern

Methods

n/a	Involved in the study	
☒	☐	ChIP-seq
☒	☐	Flow cytometry
☐	☒	MRI-based neuroimaging

Human research participants

Policy information about studies involving human research participants

Population characteristics	healthy participants, mean age = 27, 13 females
Recruitment	Participants were students at Freie Universität Berlin. The study was about basic vision, so any selection bias should not influence the results.
Ethics oversight	Freie Universität Berlin

Note that full information on the approval of the study protocol must also be provided in the manuscript.

Magnetic resonance imaging

Experimental design

Design type	event-related design
Design specifications	Stimuli were presented using a rapid event-related design (stimulus duration, 500 ms) while participants performed a fixation-cross-brightness-change detection task and their brain activity was measured with a 3T fMRI scanner. Stimuli were overlaid with a light grey fixation cross and displayed at a width of 4° visual angle. Each image was presented once per run in random order (9-13 runs in total). Each run contained 32 randomly timed null trials without stimulus presentation.

Behavioral performance measures	Participants had to report a short (100 ms) change in the luminance of the fixation cross via button press (mean performance 97% (+/- 0.14, standard error)).

Acquisition

Imaging type(s)	fMRI
Field strength	3T
Sequence & imaging parameters	Magnetic resonance imaging was acquired using Siemens 3T Trio with a 12-channel head coil. For structural images, we used a standard T1-weighted sequence (176 slices). The TR was 2 s and the ITI was 3 s. For fMRI, we conducted 9–13 runs in which 249 volumes were acquired for each participant.
Area of acquisition	whole brain

Diffusion MRI ☐ Used ☒ Not used

Preprocessing

Preprocessing software	The fMRI data were preprocessed using SPM12 (https://www.fil.ion.ucl.ac.uk/spm/). For each participant and session separately, functional data were spatially realigned, slice-time corrected, and coregistered to the participant-individual T1 structural image.
Normalization	no normalization for ROI, normalization for searchlight analysis
Normalization template	SPM12 template
Noise and artifact removal	We estimated the fMRI responses to the 128 image conditions with a general linear model, which included movement parameters as nuisance terms.
Volume censoring	did not perform volume censoring

Statistical modeling & inference

Model type and settings	multivariate, Representational Similarity Analysis
Effect(s) tested	no ANOVA or factorial designs tested

Specify type of analysis: ☐ Whole brain ☒ ROI-based ☐ Both

Anatomical location(s): Probabilistic brain atlas (Wang et al., 2015)

Statistic type for inference (See Eklund et al. 2016)	voxel-wise
Correction	FDR correction

Models & analysis

n/a	Involved in the study
☒	☐ Functional and/or effective connectivity
☒	☐ Graph analysis
☒	☐ Multivariate modeling or predictive analysis
